# Anti-carcinogenic and anti-angiogenic properties of the extracts of *Acorus calamus* on gastric cancer cells

**Published:** 2017

**Authors:** Samaneh Rahamooz Haghighi, Malek Hossein Asadi, Hassan Akrami, Amin Baghizadeh

**Affiliations:** 1 *Department of Plant Breeding, Faculty of Sciences and Modern Technologies, Graduate University of Advanced Technology, Kerman, Iran*; 2 *Department of Biotechnology, Institute of Science and High Technology and Environmental Sciences, Graduate University of Advanced Technology, Kerman, Iran*; 3 *Department of Biology, Faculty of Science, Razi University, Kermanshah, Iran*

**Keywords:** Acorus calamus, Gastric cancer, Anti-proliferation, Angiogenesis, Oct4, Nucleostemin

## Abstract

**Objective:**

*Acorus calamus* (*A. calamus*) has been used as a medicinal plant in Asia for its effects on digestive system for the last 2000 years. To investigate the anti-cancer activity of rhizome of* A. calamus*, the ethanolic and methanolic extracts and essential oil of the rhizome were prepared and their effects were assessed on human gastric cancer cell line (AGS).

**Materials and Methods:**

The viability of cells which were treated with the extracts and the essential oil was assessed by MTT assay. To evaluate the anti-angiogenic property of the extracts, *in vitro* tube formation assay was done. Cell cycle distribution and the expression of Oct4 and Nucleostemin, after treatments, were checked by flowcytometry and quantitative RT-PCR, respectively. Furthermore, analysis of essential oil from *A.calamus* was done by GC-MS.

**Results:**

Our results showed that the growth of AGS cells was inhibited by the extracts and essential oil and the extracts inhibited the angiogenesis in HUVEC cells. Our data revealed that the extracts and essential oil of *A. calamus* caused G1 arrest in AGS cells and downregulation of Oct4 and NS after treatment. By GC-MS analysis, we found new compounds such as epiprezizaene, valencene and isocyclocitral in essential oil of *A. *

**Conclusion:**

All together, our results showed that the extracts of *A. calamus* have anti-proliferative and anti-angiogenic effects on cancer cells.

## Introduction

In 2008, the global incidence of newly diagnosed cancer cases and cancer-associated deaths were reported to be approximately 12.7 million and 7.6 million, respectively. Stomach or gastric cancer is known as the fourth most common malignancy. This cancer causes the death of 738,000 people annually and is known as the main culprit in the death of 10% of all cancer-related mortality worldwide. (Jemal et al., 2011 ▶). It is estimated that over 70% of people diagnosed with or killed by stomach cancer inhabit the developing countries. Gastric cancer also exhibits a higher rate of incidence in Iran and is recognized as one of the major causes of cancer-associated mortality in the country. (Malekzadeh et al., 2009 ▶). Therefore, it seems necessary to find new and more effective drugs for cancer therapy. The rate of death by cancers and the destructive side effects of chemical anti-cancer drugs are the major reasons which motivated the researchers to find new, more effective anti-cancer drugs with fewer side effects. 

One of the most important sources of new drugs are herbal medicine and in this regard, attention on plant components which have anti-cancer properties has been increased (Dorai and Aggarwal, 2004 ▶; Gharaei et al., 2013 ▶). Herbal medicines, also called botanical medicines, play significant roles in the treatment of various diseases. These compounds have emerged as one of the most efficient alternative candidates for the development of anti-cancer therapies. Current information provides support for the notion that traditional medicine is exploited by about 80% of the world population. Traditional medicine is mainly based on plant-derived substances which possess a variety of primary health care properties. (Balakumbahan et al., 2010 ▶). Medicinal plants possess secondary metabolites which are the main sources of medicinal drugs having curative nature and efficiency in human health care. It is believed that phytochemicals which are obtained from the medicinal plants have many benefits for human health and in recent years they have attracted attention because of their efficacy and cost effectiveness (Dahiru et al., 2006 ▶).

*Acorus calamus* (*A. calamus*) which is also known as *sweet flag* (Araceae family) (Meena et al., 2010 ▶) is a beneficial and medicinal plant that has been used in herbal therapies and human health care preparations in the Asia for a long time (Singh et al., 2011 ▶). 

The origin of *A.calamus* is thought to be India, through now it is also found in other parts of the world including Asia Minor, Sri Lanka, Burma, Japan, China, southern Russia, Europe, and northern USA. This plant is a perennial plant and was spread outside its native area in Asia (Balakumbahan et al., 2010 ▶). The exploitation of *A.calamus* that is cultivated in Baluchestan and Kerman provinces of Iran has revitalized interest in traditional medicine. In addition to pharmaceutical properties of *A. calamus* in treatment of many diseases, it is valuable in food and perfume industry. For instance, its essential oil is used in perfume industry, as a flavor for pipe tobacco and in tea, and in Europe, it is added to wine. Also, its root is one of the potential additives of absinthe (Choi et al., 2013 ▶). The inner part of the young stems makes the salads very delicious and the dried and powdered rhizome is a suitable alternative for cinnamon, nutmeg and ginger (Kumar et al., 2014 ▶; Marcy et al., 2005 ▶).

*A.calamus* possesses a long history of use for healing purposes in India and China. (Mukherjee et al., 2007 ▶). In India, *A.calamus* have been applied to treat gastric problems, stomach cramps, and cancer (Gaidhani et al., 2009 ▶), as well as to inhibit the side effects caused by hallucinogens (Devi and Ganjewala, 2011 ▶). *A.calamus* is known as an important plant in Ayurvedic medicine in that it is viewed as a “rejuvenator” for the nervous system and as a therapeutic agent for digestive disorders. *A.calamus* has been referred to as a “wonder drug” in popular European books on medicinal herbs and frequently exploited as a nervine in folk medicine which results from its tranquilizing effects of cis-isoasarone. (Meena et al., 2010 ▶).* A. calamus* extract has been used in traditional Chinese prescription due to its beneficial effects on memory and learning performance; also, anti-aging effect in senescence has been reported for this plant (Nishiyama et al., 1994 ▶; Palani et al., 2009 ▶; Zhang et al., 1994 ▶). Various extracts of *A. calamus* have anti-diabetes, anti-proliferative, immunosuppressive, hypolipidemic and anti-carcinogenic effects and the rhizomes and leaves were found to possess anti-carcinogenic activity in human lymphocytes (Mehrotra et al., 2003 ▶). 

Ethanolic extract of rhizome of this plant possesses sedative, analgesic, moderately hypotensive and respiratory depressant properties (Agarwal et al., 1956 ▶). Many other activities have been reported from this plant including, anti-fungal (Rajput and Karuppayil, 2013 ▶), anti-bacterial ( Rahamoz- Haghighi et al., 2014 ▶) and anti-inflammatory effects (Kim et al., 2012 ▶; Tiwari et al., 2010 ▶).

Despite the beneficial role of this plant against many ailments, its anti-cancer properties are not well understood. Thus, to elucidate the anti-cancer properties of *A. calamus*, we investigated the potential anti-proliferative activity of ethanolic and methanolic extracts and its essential oil on human gastric cancer cell line (AGS) in comparison with human fibroblast cells (HSkMC) as normal cells. Furthermore, the effects of alcoholic extracts on tube formation of HUVEC cells were investigated. GC-MS analysis was performed for identification of bioactive compounds of essential oil of *A. calamus*. 

## Materials and Methods

**Preparation of alcoholic extracts**

The rhizome of *A. calamus* (Accession No: UNA00026156) was bought from a local market of Kerman province of Iran and authenticated at the Botany Department, Kerman University, Kerman, Iran. 20 g of the dried rhizome derived from the plant *A.calamus* was grounded and changed into a coarse powder. The powder was put in a Soxhlet extractor harboring 500 mL of each of either methanol or ethanol and kept for 8 hours. This procedure was repeated three times. The extracts were concentrated within a rotary evaporator for 75 min (vacuum condition, 45°C) and then stored at 4 °C. The extracts of *A. calamus *were dissolved in dimethyl sulphoxide (DMSO) for preparation of different concentrations (15, 30, 60, 120, 240 and 480µg/ml). 

**Essential oil preparation**

100 g of the shade-dried rhizome obtained from *A.calamus* was grounded and changed into a coarse powder. The powder was exploited for hydrodistillation using a clevenger’s apparatus for 3 h. This procedure was required to obtain the volatile constituents of the powder as essential oils. The desiccation of essential oils was conducted over anhydrous sulfate sodium. The dried oil was held in sealed clean glass vials at 4 °C. To make different concentrations, the essential oil was dissolved in culture media (15, 30, 60, 120, 240 and 480 µg/ml).

**The gastric adenocarcinoma cell (AGS) and fibroblast cell (HSkMC) culture**

The gastric adenocarcinoma cell line (AGS) and fibroblast cell line (HSkMC) were obtained from national cell bank of Iran (Pasteur institute, Iran, Tehran) and were cultured at 37°C in a humidified atmosphere of 5% CO_2_ in RPMI1640 medium (Gibco, USA), supplemented with penicillin, streptomycin (100 U/ml and 100 µg/ml, respectively) and 10% fetal bovine serum. 

**Viability assay**

The inhibitory effect of the ethanolic and methanolic extracts and essential oil of *A.calamus* on human gastric cancer and fibroblast cells was determined by MTT assay. MTT assay is based on the enzymatic reduction of the tetrazolium salt MTT in viable/metabolically active cells. Cells were seeded at a density of 2×10^5^ cells/well in a 96-well plate. The cells were allowed to attach and grow for 24 hr. In order to evaluate the growth inhibitory effects of* A. calamus*, cells were treated with various concentrations (15, 30, 60, 120, 240 and 480 µg/ml) of alcoholic extracts and essential oil and incubated for 1 to 3 days at 37 °C in a 5% CO_2_ humidified atmosphere. The cells that were incubated with 5-FU (Austria, Ebewepharma), a commonly used anti-cancer drug, served as positive control and the ones treated with DMSO were used as negative control. One , two and three days after treatment, 20 µl of 5 mg/ml MTT solution was added to cells and incubated at 37 °C for 4 hr. After this period, the medium was removed and 200 µl DMSO was added to each well to dissolve the formazan crystals. The absorbance of formazan dye was read using an ELISA plate reader at 490 and 630 nm and the optical density (OD) was recorded. All of the above-mentioned steps were performed thrice. The following formula was used to calculate the inhibitory rate of cell growth: Growth inhibition% = (1 - OD extract treated) / OD negative control × 100

**Cell cycle analysis**

The cells were rinsed with phosphate buffered saline (PBS) and then dissociated using 0.025% trypsin-EDTA. The separation of cells from each other leads to the appearance of single cell suspensions. The dissociated cells were exposed to washing with the growth medium and then stained with propidium iodide solution (50 μg/mL). The staining solution contained 0.1% triton X-100 and sodium citrate. (Asadi et al, 2011) and the single cell suspensions were used for flowcytometric analysis (Partec, Germany). Cell cycle profiles were analyzed using Partec Flomax software.

**RNA extraction, reverse transcription and Real-time PCR**

The isolation of total RNA was carried out using RNX plus solution following the manufacturer’s instructions. Total RNA was treated with RNAse-free DNase (Fermentas, Lithuania). The following components were used to synthesize the first strand of complementary DNA (cDNA): 1 μg of template RNA, 200 U/μL of MMLV reverse transcriptase (Fermentase, Lithuania), 20 U of RNase inhibitor, and dNTP mix (final concentration of 1 mM) with oligo (dT) 18 priming. The final reaction volume was 20 μL. The first strand synthesis was performed at 42°C for 60 min. Specific primers and probes were designed for Nucleostemin (NS), GAPDH and Oct4 using AlleleID 4.0 and Genrunner software (Table1).

**Table 1:** The sequence of designed primers

***Genes***	***Designed oligo***	***Sequence***	***Amplican length***
***Nucleostemin***	*F*	ATAGCATCCTTTTCCAGTCTTCCG	*118*
	*R*	TCTTTGTCATCCTCCCCTCTCC	
***GAPDH***	*F*	GTGACCATGAGAAGTATGACAAC	*123*
	*R*	CATGAGTCCTTCCACGATACC	
***Oct4***	*F*	GAGAATTTGTTCCTGCAGTGC	*470*
	*R*	GTTCCCAATTCCTTCCTTAGTG	

Real-time PCR reactions were carried out by using 2 μl of the first cDNA strand, 9 μl of Sybergreen solution (Takara), 0.8 μl of 20 pmol of each primer in the final volumes of 25 μl. The PCR carried

out under the following conditions: initial denaturation at 94 °C for 4 min, a 30 cycle amplification (94 °C for 10 S, 58.5 °C for 15 sec, 72 °C for 30 sec), and a final extension at 72 °C for 2 min.

All experiments were conducted in duplicate or triplicate. The REST 2008 software (Relative Expression Software Tool, V2.0.7, Corbett Research Pty. Ltd) was exploited to conduct group-wise comparisons and analyze statistically the results of gene expression. 

***In vitro*** **angiogenesis assay**

The tube formation assay was performed by ECM gel (Sigma-Aldrich) according to the manufacturer’s protocol. In brief, 100 µl of ECM gel was added to each well of 24-well plates and incubated at 37 °C for 2 hr to solidify ECM gel. HUVEC cells were cultured on the solidified ECM gel at the concentration of 3-4 × 10^4^ cells in DMEM: F12 (1:1) containing 5% FBS, treated with 120 µg/ml of the extracts and incubated at 37 °C, 5% CO_2_ and 95% humidity air for 24 hr. The tube formation was observed under an inverted microscope (CETI, UK). 

**Gas chromatography-Mass spectrometry analysis**

Gas chromatography–mass spectrometry analysis of the *A. calamus *essential oil was performed on 0.1 µl of the pure oil sample and subjected to GC-MS analysis. The apparatus used for analysis had the following specifications: Agilent Technology GC 7890A, MS 597SC, capillary column (30 m × 250 µm × 0.25 µm, ID=0.32 mm). Helium was used as the carrier gas (1.0 ml/min), the temperature of injector was 250 °C, and the temperature of oven changed from 50 °C to 260 °C (with an increase rate of 5 °C/min). The oven temperature was finally kept isothermal with chemical constituents of *A. calamus* oil for 52 min. GC-MS analysis on the oil derived from *A.calamus* showed that terpenoid compounds are the oil major constituents.

**Statistical analysis**

All experiments were conducted in triplicate and group-wise comparison and statistical analysis of results were carried out by ANOVA and Duncan tests. SPSS program version 20 was utilized for statistical analyses and p<0.05 was considered as statistically significant.

## Results

**Growth-inhibitory effect of the extracts and essential oil**

**Figure 1:**
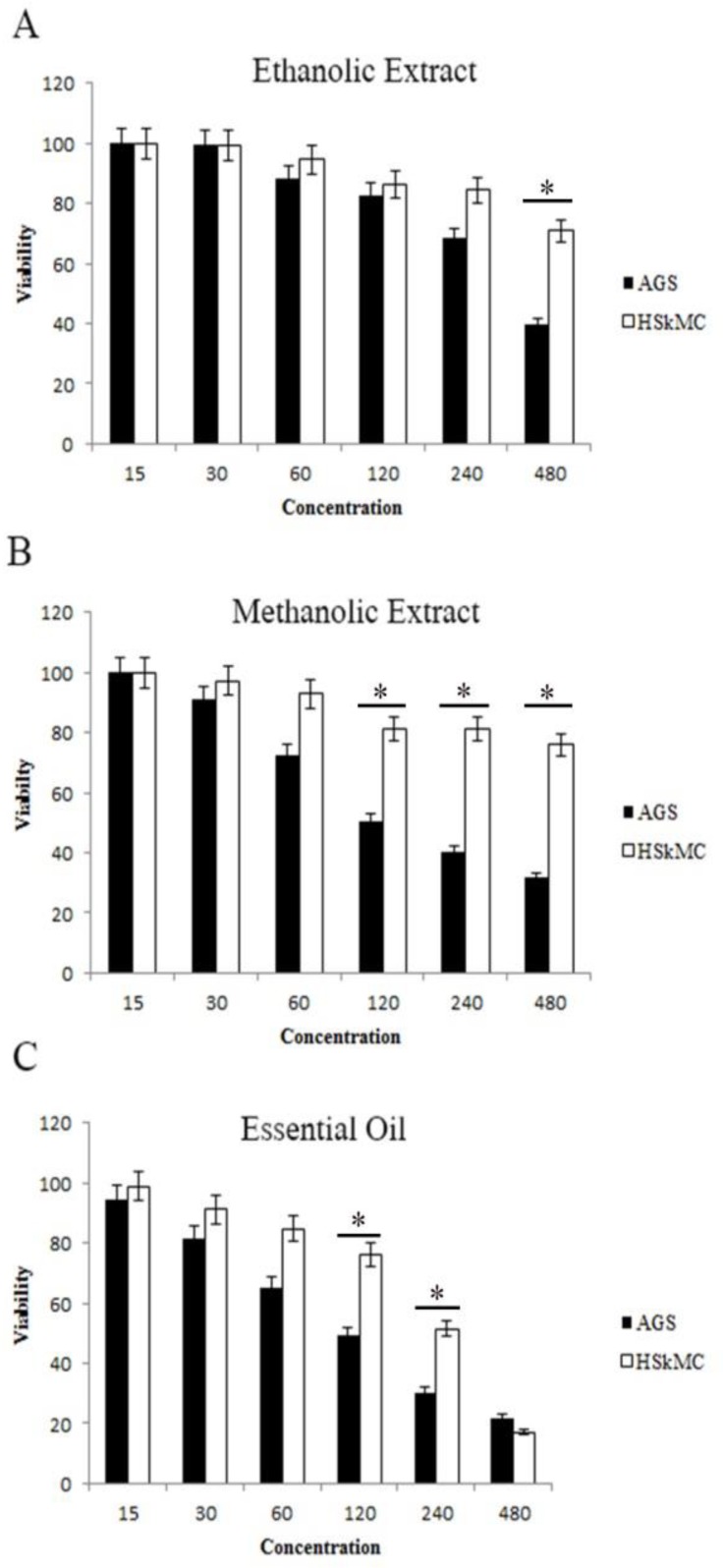
Gastric cancer (AGS) and human fibroblast (HSKMC) cells were treated with the ethanolic and methanolic extracts of *A. calamus* and also its essential oil and harvested for MTT assay. A) Ethanolic extract showed significant anti-proliferation effect on AGS cells at the concentration of 480 µg/ml (p<0.05). B) Methanolic extract showed significant anti-proliferative effect on cancer cells at the concentration of 120 µg/ml (p<0.05). C) Essential oil of *A.calamus* has inhibitory effect on cancer cells at 120µg/ml concentration, similar to the effect of 5-FU (conventional anti-cancer drug) (p<0.05). Values shown represent the mean ± SEM. The asterisk shows statistical significant differences

Results of MTT assay showed that the methanolic and ethanolic extracts and essential oil of *A. calamus* rhizome have cytotoxic effect on AGS cells and their components might act against the proliferation of AGS cells. There was a significant concentration and time-dependent decrease in the proliferation of AGS cancer cells after treatment with the extracts and essential oil of *A. calamus* (Figure 1).

The ethanolic extract of *A. calamus* had no cytotoxic effect on AGS and HSkMC cells except at 480 µg/ml (Figure 1A) but the methanolic extract had a significant cytotoxic effect at 120 µg/ml after 48 hr of treatment on AGS cells while it had no significant effect on HSkMC cells (Figure 1B). It must be mentioned that the cytotoxic effect of methanolic extract on AGS cells increased along with increasing concentrations of the extract (Figure 1B) The essential oil of *A. calamus* has significant effect on AGS cells at 120µg/ml after 48 hr of treatment, similar to the effect of 5-FU (Figure 1C).

**Cell cycle alterations following treatment of cells with extracts and essential oil**

**Figure 2:**
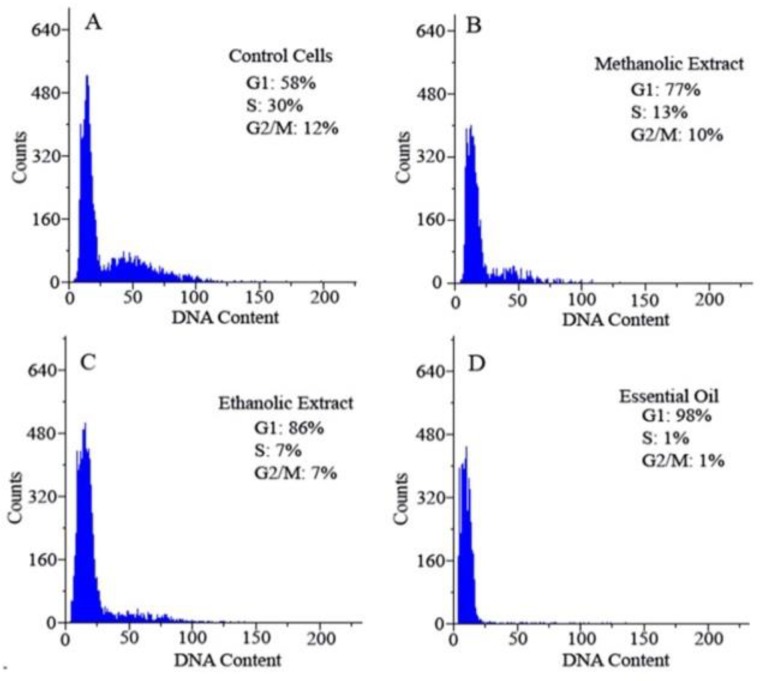
The effect of alcoholic extracts of *A. calamus* on cell cycle distribution of AGS cells. The cell cycle distribution in the absence of the extracts and essential oil (A) compared to cells which were treated with methanolic extract (B), ethanolic extract (C) and essential oil (D). After treatment, we observed G1 arrest in cell cycle

Cell cycle distribution of the AGS cells was determined by flowcytometry after treatment with alcoholic extracts and essential oil of *A. calamus*, for 48 hr. Our results showed that AGS cells treated with the extracts and essential oil arrested in G1 phase of cell cycle in comparison to control cells (Figure 2).

**Anti-angigogenic property of the extracts and essential oil**

**Figure 3:**
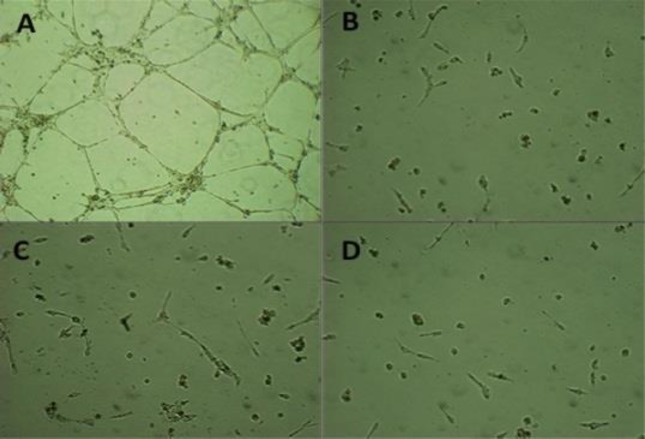
The tube formation assay was conducted by HUVECs which were cultured on fibrin matrices in the absence (**A**) or presence of the methanolic (B), and ethanloic (C) extracts and essential oil (D). After 24 hr, light microscopy images of cells showed that the methanolic extracts inhibited the angiogenesis

HUVECs seeded on an ECM gel were treated with the methanolic and ethanolic extracts of *A. calamus* at different concentrations (30, 60, 120, 240 and 480 µg/ml) for 24 hr. Our results revealed that inhibition of formation of tube-like structures occurred at 120 µg/ml of methanolic extract and 240 µg/ml of ethanolic extract compared to control cells (Figure 3).

**Downregulation of Oct4 and Nucleostemin after treatment of the cells with the extracts**

**Figure 4:**
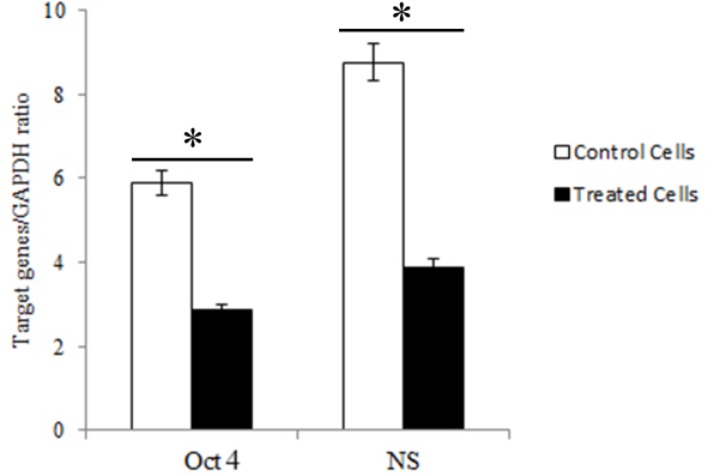
*Oct4* and Nucleostemin, highly expressed factors in gastric cancer, were downregulated in cancer cells after treatment with the alcoholic extracts of *A. calamus *(p<0.05). Data are presented as mean ± SEM. The asterisk shows statistical significant differences

Based on our previous studies which showed the *Oct4 *and Nucleostemin (*NS*) were over-expressed and had causative role in tumorgenesis in gastric cancer. We assessed their gene expression in AGS, a gastric cancer cell lines after treatment with methanolic extracts of *A. calamus*. Our data showed that *Oct4* and *NS* were significantly downregulated after treatment of the gastric cancer cells with the extract of *A. calamus* (Figure 4).

**Determination of the composition of essential oil from** ***A. calamus*** **by GC-MS**

**Figure 5:**
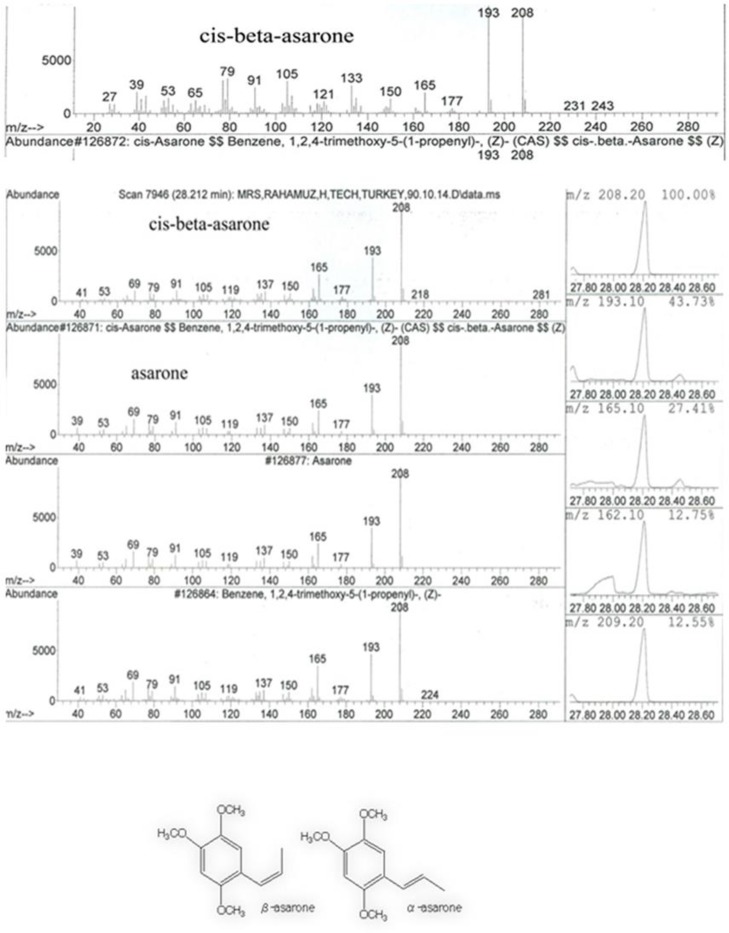
GC-MS diagram showing the peak of cis-beta- asarone, one of the important bioactive compound present in essential oil of *A. calamus*

The composition of the essential oil of *A. calamus* was analyzed by GC-MS and its components were identified according to Wiley7n.1 library. Different parts of the plant showed the presence of a large number of phenyl propanoids, monoterpenes and sesquiterpenes as isomers of asarone (Figure 4). Study of the volatile components using GC-MS detected 47 peaks in the analysis of essential oil of *A. calamus* with dehydroxy-isocalamendiol (14.73%), Anethole (12.22%) and shyobunone (7.57%) as the dominant constituents. Our present study showed that *A. calamus* essential oil has anti-cancer compounds because we observed that these compounds in the main peaks at 19.788; 14.09; 27.219;22.574; 23.994, 24.879; 11.621 and 27.725 min (Table 2). Furthermore, according to the library database, the spectra of the compounds matched with the anethole, linalool, spathulenol, beta-elemene, (-)-epiprezizaene, ar-curcumene, limonene and β-asarone spectra (Figure 5). Compared to previous studies, in the essential oil, we found new compounds such as epiprezizaene, alpha-curcumene, valencene and isocyclocitral.

**Table 2:** Bioactive components of essential oil of *Acorus calamus* determined by GC- MS

**Peak number**	**Compound**	**Time**	**Area**
**1**	Camphene	8.71	0.58%
**2**	o-cymene, o-cymol and p-Cymene	11.50	4.75%
**3**	Limonene, dl-limonene	11.62	0.40%
**4**	Gamma-terpinene	12.69	3.80%
**5**	alpha-terpinolene, L-linalool, β-Linalool	14.09	0.98%
**6**	Camphor	15.48	3.36%
**7**	3cyclohexen-1-ol,4-methyl-1-(1-meth…) or Terpinenol-4-ol	16.49	0.19%
**8**	Estragole	17.16	2.35%
**9**	Carvacrol	17.80	3.08%
**10**	2-cyclohexan-1-one,2-methyl-5-(1-methye..) and ((S)-Carvone)	18.49	0.26%
**11**	Cinnamaldehyde	19.26	0.21%
**12**	Anethole, trans-anethole	19.78	12.22%
**13**	Thymol	19.88	1.87%
**14**	Eugenol	21.64	0.30%
**15**	1h-3a 7-methanoazulene or α-Cedrene	22.31	0.20%
**16**	Beta-elemene	22.57	0.42%
**17**	Naphthalene, 1,2,3,4,4a,5,6,8a-octahydro-7-or γ-Cadinene and β-Cedrene	23.16	1.35%
**18**	(-)-aristolene, Aristolene or Aristolen	23.29	2.10%
**19**	1(5),3-aromadenedradiene and 5-n-butyltetralin and 4,5-dehydro-isolongifolene	23.52	0.23%
**20**	Calarene	23.64	3.10%
**21**	(-)-5-epiprezizaene and (-)-piprezizaene	23.99	0.42%
**22**	(-)-5-epiprezizaene and (-)-piprezizaene	24.12	1.90%
**23**	Isohomogenol, trans-methyl isoeugenol and cis-methyl isoeugenol	24.23	0.73%
**24**	(-)-beta-acoradiene and gamma-curcumene	24.48	0.21%
**25**	Aristolene and Zingiberene	24.67	0.42%
**26**	benzene,1-(1,5-dimethyl-4-hexenyl)-4-methyl- or Alpha-Curcumene	24.87	0.41%
**27**	Valencene l and α-Gurgujene	25.02	1.67%
**28**	Alpha-selinene	25.19	3.78%
**29**	cyclohexanone,3-ethenyl-3-methyl-2-(1-methylethenyl)-6-(.. Or shyobunone	25.73	7.57%
**30**	delta-cadinene and beta-cadinene	25.89	1.03%
**31**	2-hexyl-1-decan-3-yne and 2 ,5-cyclohexadiene-1 4-dione and 2 6-bis(1 1-dimethylethyl)-4a…	26.09	5.78%
**32**	Calacorene and 5,8-dimethylisoquinoline	26.37	1.21%
**33**	cyclohexanemethanol,4-ethenyl-alpha,alpha,4-trimethyl-3-…and Elemol	26.51	0.20%
**34**	Calacorene and alpha-calacorene	26.84	0.87%
**35**	1h-cycloprop[e]azulen-7-ol,decahydro--… or Spathulenol, (+)spathulenol and (-)-spathulenol	27.21	0.91%
**36**	isoaromadendrene epoxide and vulgarol B and Junipene or longifolene	27.33	0.52%
**37**	trans-isoelemicin and cis-asarone or β-asarone and Isoelemicin	27.72	0.23%
**38**	dehydroxy-isocalamendiol	27.99	14.73%
**39**	cis-asarone or Asarone- trans	28.21	3.37%
**40**	2,5-diethyl-3,6-dipropylpyrazine and 5-methylene-10-oxo-10,…and 4a,5,8,8a.beta-tetrahydro-2-methoxy-4a...,	28.45	2.69%
**41**	1-t-buty-2,4-dimethyl-1-cyclohexene and 3,4-dihydro-6-fluorocoumarin and spiro[4.5]dec-8-en-7-ol,4,8-dim…	28.78	1.08%
**42**	Valencene	28.97	0.36%
**43**	Isocyclocitral	29.12	0.64%
**44**	spiro[4.5]decan-7-one,1,8-dimethyl-…, 1-t-buty-2,4-dimethyl-1-cyclohexene and 3,4-dihydro-6-fluorocoumarin	29.32	0.42%
**45**	(-)-isoledene	29.63	1.14%
**46**	1,4-cis-1,7-cis-acorenone , 1,4-cis-1,7-trans-acorenone	29.80	5.68%
**47**	Isocalamendiol	30.92	0.28%

## Discussion

A wide variety of secondary metabolites in plants are used as drugs for cancer treatment because they have anti-proliferative properties (Gali-Muhtasib and Bakkar, 2002 ▶). Most of the chemical cytotoxic drugs using for cancer treatment, have side effects. So, it is necessary to find new drugs with high efficiency and low side effects (Powis, 1983 ▶). Recently, many drugs which have been obtained from plant sources such as currcumin, artisimin and taxol are used for cancer treatment (Rajkumar et al., 2009 ▶). Due to its various uses, there has been a growing demand for effective compounds of plants. The presence of considerable level of polyphenolic compounds including flavonoids, flavonols, proanthocyanidines suggested that plant parts may have antioxidant activity. Extracts of different parts of *A. calamus* and *calamus* oil are now widely used in pharmaceuticals and traditional systems of medicines for a number of ailments. 

In the first phase of our study, the cytotoxic activity of the extracts and essential oil of *A. calamus* rhizome was investigated by MTT assay on gastric cancer cell line (AGS) and human fibroblast cells (HSkMC) as normal cells. Our results showed that the ethanolic and methanolic extracts have significant anti-proliferative effect on AGS cells. Methanolic extract of the plant showed higher anti-proliferative effect in comparison to ethanolic extract. Based on our results, we suggest that the metabolites which are solved in methanol have higher cytotoxic effect in comparison to the ones which are soluble in ethanol.

The US National Cancer Institute argues that the IC50 value has to be below 30µg/ml so that a crude extract can be regarded appropriate for further purification (Rajkumar et al., 2009 ▶). Essential oil of rhizome of *A. calamus* also inhibits the proliferation of cancer cells at 120µg/ml, similar to that of 5FU (conventional anti-cancer drug). Although our recorded effective dose is higher than the standard dose, but it is still noticeable. So, it may be concluded that the essential oil potentially has medicinal value. Both extracts and essential oil have significant cytotoxic effects on cancer cells in comparison to normal cells following 48 hr of treatment. As we know, the essential oils of the plant has toxic products and at high concentrations it can destroy cells, so it should be noted that low concentrations of essential oil have the same effect as high concentrations of extracts. In this context, the extracts derived from the rhizome of *A.calamus* might be regarded as a valuable source of metabolites with potential application as anti-tumor drug precursors. Furthermore, we performed GC-MS to investigate the components of essential oil of rhizome of *A. calamus*. GC-MS analysis of essential oil from *A. calamus* rhizome showed the existence of phenyl propanoids, monoterpenes and sesquiterpenes such as isomers of asarone. Based on our results from GC-MS of the essential oil, we detected 47 peaks in the analysis of the essential oil with dehydroxy-isocalamendiol (14.73%), Anethole (12.22%) and shyobunone (7.57%) as the dominant constituents. In spite of the pervious components which were reported to be present in the essential oil of *A. calamus* rhizome, we observed new compounds in the essential oil such as epiprezizaene, alpha-curcumene, valencene and isocyclocitral. 

In the next phase of our study, we assessed the cell cycle distribution of AGS cells treated with rhizome extracts and essential oil. Our results revealed that the extracts and essential oil cause G1 arrest in AGS cells. Based on our data, the rhizome extracts and essential oil may have medicinal value in cancer therapy because they induced G1 arrest in cancer cells that were prolapsed from the normal cell cycle.

Angiogenesis is a procedure through which new blood vessels are formed. This process provides cancer cells with oxygen and nutrients, thereby being considered as a prerequisite for the growth of tumor cells. The angiogenic processes play leading roles in the progression, invasion, and metastasis of neoplastic cells and are typically considered as prognostic indicators for tumors. Therefore, targeting tumor angiogenesis is of great importance. So, the effect of the rhizome extracts on the angiogenesis process was evaluated. Our results revealed that the extracts inhibited the formation of tube-like structures as compared to control cells. 

The therapeutic properties of the *A.calamus* essential oil can be attributed to the synergistic function of its various components. However, our present study showed that the ethanolic and methanolic extracts and essential oil of *A.calamus* rhizome significantly inhibited the proliferation of human gastric cancer cells and they acted in a dose and time-dependent manner. The higher concentration of extracts and the longer time of the treatment have more significant cytotoxicity effect. Therefore, based on our results, we suggested that the rhizome extracts and essential oil have anti-proliferative effects on gastric cancer cells but no significant growth inhibitory effects were observed on normal cells. So, they could be used as anti-cancer drugs against tumor growth and propagation because they have potent anti-cancer components. Taken together, our findings support the notion that the rhizome of *A.calamus* might contain a variety of secondary metabolites that represent potential to be used for the development of anti-tumor drug precursors. The purification of these bioactive compounds is thought to be helpful for the formulation of therapeutic agents against cancer. 
